# Proteostasis in T cell aging

**DOI:** 10.1016/j.smim.2023.101838

**Published:** 2023-11

**Authors:** A. Elisabeth Gressler, Houfu Leng, Heidi Zinecker, Anna Katharina Simon

**Affiliations:** a Max-Delbrück-Center for Molecular Medicine in the Helmholtz Association (MDC), Robert-Rössle-Str. 10, 13125 Berlin, Germany; b Kennedy Institute of Rheumatology, University of Oxford, Roosevelt Drive, Oxford OX3 7FY, United Kingdom; c Department of Biological Chemistry, David Geffen School of Medicine, University of California, Los Angeles (UCLA), Los Angeles, CA 90095, USA; d Ascenion GmbH, Am Zirkus 1, Bertold-Brecht-Platz 3, 10117 Berlin, Germany

**Keywords:** Proteostasis, T cell, Aging, Translation, Degradation, Inflamm-aging

## Abstract

Aging leads to a decline in immune cell function, which leaves the organism vulnerable to infections and age-related multimorbidities. One major player of the adaptive immune response are T cells, and recent studies argue for a major role of disturbed proteostasis contributing to reduced function of these cells upon aging. Proteostasis refers to the state of a healthy, balanced proteome in the cell and is influenced by synthesis (translation), maintenance and quality control of proteins, as well as degradation of damaged or unwanted proteins by the proteasome, autophagy, lysosome and cytoplasmic enzymes. This review focuses on molecular processes impacting on proteostasis in T cells, and specifically functional or quantitative changes of each of these upon aging. Importantly, we describe the biological consequences of compromised proteostasis in T cells, which range from impaired T cell activation and function to enhancement of inflamm-aging by aged T cells. Finally, approaches to improve proteostasis and thus rejuvenate aged T cells through pharmacological or physical interventions are discussed.

## 1 Introduction

Immune aging, including dysregulation of the adaptive immune response that relies on T and B cells, significantly contributes to the aetiology of various age-related pathologies such as neurodegenerative disorders, cancer and cardiovascular conditions. The growing high proportion of older people worldwide thus embodies a challenge to health care systems around the world.

With age, progressive systemic chronic inflammation combined with cumulative oxidative stress, e.g. due to mitochondrial dysfunction, and persistent environmental insults lead to intensified dysregulation of immunity. Particularly, the decline in adaptive immune responses is caused by T cell aging, with CD4^+^ and CD8^+^ T cells deteriorating in function, accompanied by changes in cell number, proliferation and differentiation [1], [2]. Reduced T cell function and maintenance occurs in naïve, effector and memory T cells upon aging. Naïve T cells arise from the thymus or through homeostatic proliferation in the periphery, and the contribution of the latter to the maintenance of peripheral naïve T cells is higher in humans compared to mice [3], [4], [5]. Reduced frequencies of circulating naïve CD8^+^ T cells are observed in older humans [6], [7], and this reduction is considered a hallmark of aging [8]. In addition, older adults show an increase in susceptibility to infectious diseases and exhibit less effective vaccine responses due to diminished generation of a memory CD8^+^ T cell pool which is key to mediate protective immune reactions to new antigens [9]. The population of naïve CD4^+^ T cells is reported to be more stable upon aging [7], however there are also studies reporting reduced frequencies in older adults [6], [10], [11], although in one study associated with a CMV-positive status [12].

**Fig. 1:**
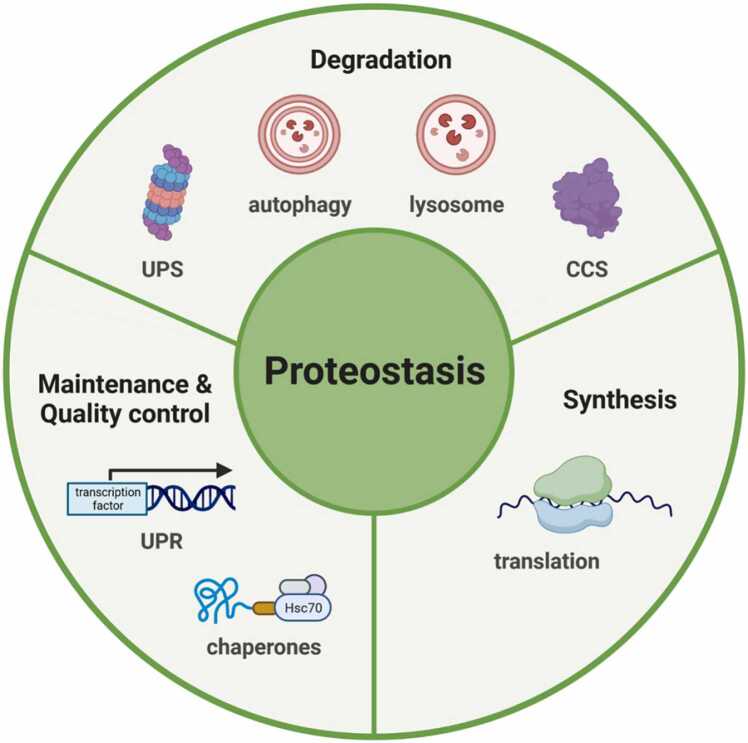
**Factors contributing to cellular proteostasis**. Maintaining a healthy proteome involves protein synthesis, maintenance and quality control, as well as degradation. Transcription and translation control the synthesis of proteins, while correct folding of proteins and maintenance are executed by the unfolded protein response (UPR) and chaperones. To recycle or degrade damaged or unwanted proteins, various cellular systems like the ubiquitin-proteasome-system (UPS), the calpain-calpastatin system (CCS), autophagy and lysosomes are employed.

A landmark publication by López-Otín *et al.* models how aging is driven by impaired function of cellular processes, highlighting loss of proteostasis as a hallmark and major cause of cellular aging [13]. Protein homeostasis (proteostasis) mediates the maintenance of a functional and balanced proteome that is fundamental for most cellular functions. The proteostasis network comprises the following various mechanisms: regulation of translation including ribosomal quality control, protein folding and quality control, and protein degradation mediated by the ubiquitin–proteasome system (UPS), autophagy and lysosomal degradation, as shown in Fig. 1 [14]. While not many studies have investigated proteostasis in T cells, some pioneering work has been published on lysosomes and autophagy. For example, increasing evidence suggests that T cell aging is caused by a failure to maintain proteostasis, which dysregulates lysosomal function and impairs T cell immune responses [15].

Autophagy, a key degradation and nutrient pathway contributing to cellular proteostasis has been demonstrated as crucial for immune cell dynamics, including differentiation and proliferation [16], [17] and compromised autophagy has recently been defined as an integrative hallmark of aging [18]. Autophagy and various other cellular processes involved in proteostasis are modulated by two cellular master regulators: mechanistic target of rapamycin complex (mTOR) and 5′ AMP-activated protein kinase (AMPK) [19]. Both mTORC1 and mTORC2 contain the protein kinase mTOR and in their active form promote protein translation, and repress autophagy and lysosomal biogenesis, while AMPK activates autophagosomal and lysosomal degradation processes [20].

Dysfunction of both autophagy and mitochondria strongly compromise the maintenance of T cell homeostasis, mainly through increased ROS production which leads to accumulation of oxidative damage in both DNA and proteins [21]. T cells with dysfunctional mitochondria show increased production of proinflammatory cytokines, potentially contributing to a process called inflamm-aging [22]. Inflamm-aging is characterized by a low-grade, persistent activation of the immune system, which in turn contributes to aging of the whole organism [23], [24]. Recently, it has been hypothesized, that CD4^+^ T cells are not only contributing to, but are in fact sufficient to drive inflamm-aging [25].

**Fig. 2:**
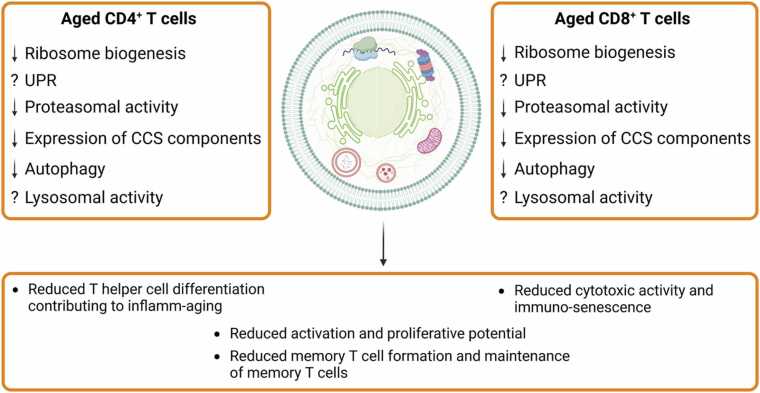
**Cellular changes in CD4**^**+**^**T cell and CD8**^**+**^**T cell affect their functionality upon aging.** Aging CD4^+^ and CD8^+^ T cells adapt to various age-related factors, like oxidative stress, protein aging and a changing cellular environment. These adaptations comprise both quantitative and functional changes, e.g. reduced abundance and impaired activity of proteins and mechanisms maintaining cellular proteostasis. This leads to a general reduction of activation and proliferation potential, as well as decreased memory T cell formation and maintenance of T memory cells for both, CD4^+^ and CD8^+^ T cell subsets, but also disturbances in CD4^+^ T cell differentiation and impaired cytotoxic activity of CD8^+^ T cells. UPR: Unfolded protein response, CCS: Calpain-calpastatin system.

In this review, we summarize current knowledge of the mechanisms by which proteostasis contributes to T cell biology and particularly T cell aging. We focus on age-related cellular changes and point to many unanswered questions that need addressing. A graphic summary of changes occurring in aged T cells and their biological consequences is given in Fig. 2. A more complete and accurate picture of the mechanisms governing proteostasis in T cells will improve our understanding of aging and generate new ideas for novel approaches to improve a healthy lifespan, not only in older adults.

## 2 How is a healthy proteome generated? – Regulation of translation

Most studies in invertebrate and vertebrate organisms, including rodents and humans have demonstrated an overall reduction of protein synthesis during aging ^reviewed in^ [26]. An age-related attenuation of the translational machinery was also reported, which leads to disrupted protein synthesis [27]. Moreover, an increased translation fidelity may contribute to a long lifespan as it is much higher in the long-lived naked mole rat cells compared to mouse cells, while there is no difference in the overall protein synthesis [28]. Translation also plays a critical role in both innate and adaptive activated immune cells [29], [30]. However, studies in T cells from aged mice or humans are lacking. Downregulation of ribosome biogenesis acts as a protective adaptation, as indicated by an increase in lifespan in yeast or *C. elegans* ^reviewed in^ [31], [32]. A downregulation of mRNA involved in pathways annotated as “ribosomal proteins” and “ribosome” were observed in aged murine naïve CD4^+^ and CD8^+^ T cells [33]. On the other hand, it has been hypothesized that with aging unstable ribosomal (r)DNA, and enlarged nucleoli may lead to increased output of rRNAs and proteins, which might contribute to overloading of the proteostastic system ^reviewed in^ [31], [34]. However, to our knowledge, studies demonstrating this concept in T cells are missing so far.

Translation is regulated by growth factors, amino acids and hormones. Insulin activates the PI3K/AKT and MAPK pathways, which regulate translation in an age-dependent manner [26]. Knockout of insulin receptors inactivates mTORC1 and thus doubles the lifespan in *C. elegans* [35]. mTORC1 signaling controls cell growth in general and has been studied extensively [36]. The precise mechanism of how mTORC1 affects the translation machinery components remains vague [37], although 4E-BP and ribosomal protein S6 kinase (p70rsk), two target proteins of mTORC1, are known to be involved [38], [39]. For example, during dietary restriction, 4E-BP, which is inactivated by mTORC1, downregulates translation, improves proteostasis and extends lifespan in flies [40], [41]. Aging brings changes to the mTOR pathway. For example, TOR1 gene deletion or mTOR downregulation leads to increased lifespan in yeast [42], worms [43], and flies [44]. In addition, a longer lifespan was also shown in mTOR and mLST8 (mTORC1 component) knockout heterozygous mice [45]. Studies on mTOR activation status in aged T cells have not given conclusive results, and as adaptations are known to occur during aging, e.g. adaptation of aged memory T cells to allow better recall responses [46], snap shot measurements might not accurately reflect mTOR status and it may vary from one T cell subtype to another. Similarly, whether mTOR inhibition is beneficial to T cells from aged organisms is still controversial. Senescent CD8^+^ T cells do not seem to enhance function after treatment with rapamycin, which inhibits mTORC1 [47]. On the other hand low dose rapaymin treatment improves memory T cell responses to vaccines in mice [48].

In response to various cellular stresses, but also during accumulation of misfolded proteins in the ER, conserved general stress-response mechanisms known as integrated stress responses are activated and result in a block of general protein translation [49], [50]. However, some mRNAs involved in cellular maintenance, repair and turnover pathways are selectively translated upon decline of global translation, and studies have shown an association of increased life-span with enhanced translation of these specific mRNAs in *C. elegans* [51] and *Drosophila melanogaster* [40]^*,* reviewed in^ [32]. Several publications have shown that the modulation of translation factors affects lifespan. For instance, in *C. elegans*, downregulation of translation factors eIF4E [52], eIF4G [51], subunits of eIF2B [53], or subunits of eIF3 [54] significantly increases the lifespan of nematodes. Although there is increasing evidence that translational control can impact T cell function in young organisms, how this applies to T cells from old organisms has not been elucidated and requires further investigation.

Recently, Elyahu *et al.* revealed, that aging promotes the polarization of CD4^+^ T cells toward regulatory and effector phenotypes [55]. In their analysis, they also found increased expression of the *Aw112010* gene in aged CD4^+^ T cells [55], which is essential for the translation of the inflammatory cytokine IL-12p40 [56] and thus could promote acute and chronic inflammation. These findings may indicate that dysregulation of translation can contribute to inflamm-aging. Up to now, there are very few studies analyzing translation in aged T cells, and thus, more functional investigation is required.

## 3 How is a healthy proteome maintained? – Chaperones and the Unfolded Protein Response (UPR)

Chaperones are essential in regulating proteostasis and preventing protein aggregation by promoting accurate polypeptide folding and translocation, ubiquitination and degradation of misfolded proteins [57], [58], [59]. Chaperones linked to the protein synthesis (CLIPS) network assist the folding of newly translated proteins [60]. In addition, the heat shock protein (HSP) network protects the proteome during oxidative stress and aging [61]. Different chaperone systems can cooperate together [62]. During aging in *C. elegans* an overload of the chaperone machinery, caused by increased protein aggregation, and an alteration of proteostasis components were observed [63]. Upregulation of chaperones in different model organisms has been associated with less protein aggregation and misfolding, and extended lifespan ^reviewed in^ [64]. Activation of T cells results in a strong increase of protein translation [65], which is accompanied by enhanced workload for the translational machinery and the chaperones promoting protein folding, and leads to potential proteotoxic stress due to the occurrence of misfolded proteins [49]. Interestingly, in young T cells, loss of the eukaryotic type II chaperonine complex protein CCT8 in mice results in defective T_H_2 cell differentiation and strongly diminishes protection against helminth infection [66]. Furthermore, CD4^+^ T cell-specific deletion of the *gp60* gene encoding Hsp90b1, a molecular chaperone and Ca^2+^ buffering protein, results in impaired activation of T cells, demonstrating the importance of ER homeostasis for T cell function [67].

ER stress in mammalian cells is sensed by three ER stress sensor proteins: PERK, IRE1, and ATF6, which initiate a mechanism known as the ER unfolded protein response (UPR^ER^) [68], [69]. Three mechanisms are employed by the UPR^ER^ to restore normal function of the cell, which are the global downregulation of translation mediated by PERK and phosphorylation of eIF2a, enabling degradation of misfolded proteins via ER-associated degradation (ERAD), and inducing signaling pathways (e.g. via ATF4, XBP1, IRE1a, ATF6), which result in increased production of molecular chaperones to assist in protein folding [70]^, reviewed in^ [68], [71], [72]. Additionally, UPR^ER^ can also act as a proapoptotic process if ER stress becomes overwhelming or UPR^ER^ is compromised ^reviewed in^ [68], [73].

Aging results in accumulation of reactive oxygen species (ROS) and nitric oxide (NO), which damage key components of the UPR^ER^ such as ER chaperones, as detected in aged liver cells [74]. This leads to decreased protein function and increased accumulation of immature and denatured proteins and thus favors proteotoxicity and cell death [74]^, reviewed in^ [72], [75]. Most studies argue for an impairment of UPR^ER^ and ER stress signaling during aging [75], [76], [77]. However, other studies demonstrated decreased, increased or unaltered expression of UPR^ER^ components, depending on organism, cell type and condition investigated ^reviewed in^ [78]. There is evidence for activation of UPR^ER^ pathways in immune cells, including T cells, during development and in mature cells [79], [80]. TCR signaling activates UPR^ER^ (PERK, IRE1a and ATF4 proteins, and splicing of XPB1 mRNA) in murine and human T cells [80], [81], [82], [83]. In human peripheral blood mononuclear cells (PBMCs), similar protein expression of HSP70 was measured in aged and young individuals, but levels of components exclusively involved in the UPR^ER^ were not quantified [84].

Given the activation of UPR^ER^ during development, priming, TCR- and infection-mediated activation of T cells, impairment of UPR^ER^ function upon aging could contribute to deficits in immune responses in aged organisms. Additionally to a role of UPR^ER^ in development and activation of T cells, there is evidence for involvement of UPR^ER^ in T cell differentiation. IRE1a has been demonstrated to promote T_H_2 effector function [83], [85], [86]. A decline of function in the UPR^ER^ upon aging could thus contribute to decreased effector functions of T_H_2 cells, favoring vulnerability to certain infectious diseases. ER stress promotes activation of UPR^ER^ and enhances T_H_17 differentiation and effector functions as demonstrated by reduced production of IL-17 after inhibition of molecules involved in the UPR^ER^ [87], [88]. Thus, a decline in UPR^ER^ function upon aging itself could also further exacerbate ER stress, thus favoring T_H_17 development and promote inflamm-aging. However, two studies report no role for IRE1a or PERK for T_H_17 differentiation [85], [89].

To our knowledge, there are no studies on the effects of aging on UPR^ER^ in CD8^+^ T cells so far. However, it is possible that a functional decline of UPR^ER^ upon aging could contribute to reduced efficiency of CD8^+^ T cells upon aging and thus increased susceptibility to infectious diseases, as UPR^ER^ is implicated in the differentiation and effector function of CD8^+^ T cells [82].

Additionally to the UPR^ER^, several studies demonstrated the importance of mitochondrial UPR (UPR^mt^) for maintenance of cellular homeostasis [90], [91]. Disturbed mitochondrial protein expression leads to transcriptional upregulation of mitochondrial, but not ER stress proteins, which in turn reduces mitochondrial protein aggregation in a mammalian cell line [92], [93]. Downstream targets of the UPR^mt^ influence ROS production, mTOR signaling, and insulin signaling, thus critically affecting cellular functions upon aging [72]. Consequently, poor mitochondrial quality control leading to mitochondrial dysfunction may contribute to increased ROS production, impaired energy supply and subsequent decline of long-lived cells ^reviewed in^ [94]. Thus, elucidating a role of UPR^mt^ in the T cell aging processes remains an interesting target of future research.

## 4 What is degraded and what is accumulating? – The ubiquitin-proteasome system (UPS), autophagy and lysosomes

One hallmark of cellular aging is the accumulation of misfolded, dysfunctional, damaged or aggregated proteins or organelles due to an overall decline in proteolytic activity [95], [96]. The accumulation of misfolded, inactive, or aggregated proteins in T cells is linked to increasing levels of ROS and ongoing Maillard reaction affecting protein function, and thus T cell maintenance, function, and survival [97], [98]. Degradation of proteins and organelles is mainly carried out by the ubiquitin-proteasome system (UPS) or the autophagosomal/lysosomal pathway, which both contribute to the prevention of cellular damage and maintenance of functionality [96], [99]. Defects in the cellular degradation pathways are implicated in aging, and enhancement of some of these pathways is connected to increasing lifespan and healthspan of various organisms [100]. Supportively, transcriptomics analysis of PBMCs revealed increased expression of genes involved in the autophagosomal-lysosomal pathway and the proteasome in cells from centenarians and their offspring compared to younger, unrelated individuals [101]. In the following paragraphs, evidence for how much each system contributes to maintenance, activation, differentiation and functionality of T cells, and how this is linked to age-related phenotypes such as inflamm-aging, is presented.

### 4.1 Ubiquitin-proteasome system (UPS)

Proteasomes are multi-subunit protein complexes, which degrade proteins marked by poly-ubiquitination in the cytosol at neutral pH, thus producing oligopeptides which can be further degraded by cytosolic peptidases into amino acids [102], [103]. There are two major forms of proteasomes, the constitutive proteasome and the immunoproteasome [104]. The 20S core unit of the constitutive proteasome consists of α-subunits, responsible for substrate recognition, and β-subunits, which provide the proteolytic activity [105]. The 20S core proteasome is often found associated with one or two 19S regulatory subunits, mediating ATP-dependent unfolding, and translocation of substrates into the catalytic 20S core unit [106]. Association of the 20S core proteasome with the 19S subunit results in formation of the 26S proteasome [106]. Exposure to IFN-γ leads to the expression of different proteasomal subunits and the formation of the immunoproteasome [107]. However, the immunoproteasome does not contribute to proteostasis overall, but is mainly involved in antigen presentation. In addition to the proteasome itself, several ubiquitin-activating, -conjugating, and -ligating enzymes, as well as deubiquitinating enzymes contribute to the initiation and regulation of proteasomal protein degradation by attachment, or detachment of ubiquitin residues to potential cargo proteins, respectively [103].

The proteolytic activity of the proteasome decreases with age in human [108], [109], [110] ^reviewed in^ [111], murine [112], and rat tissues [113], [114], as well as in flies [115], [116] ^all reviewed in^ [117]. Interestingly, decreased proteasome activity has also been found in aged human PBMCs [118], and resting and TCR-stimulated human T lymphocytes from older adults [119], [120]. Additionally, elevated proteasome abundance and activity has been demonstrated in young and old long-lived naked mole rats compared to proteasome abundance in aged mice [121], [122], which links high proteasome expression to longevity. Confirmatively, overexpression of proteasomal components prolongs the life span of *D. melongaster* [116], *C. elegans* [123], and *S. cerevisiae* [124].

Downregulation of proteasome activity upon aging could be dependent on increasing presence of post-translation modifications on proteasome subunits, as demonstrated in primary human epidermal cells [108], and in human PBMCs [118]. Various studies demonstrate modifications of proteasomal subunits as a consequence of oxidative stress either directly through oxidation [104], [125], or promotion of other modifications [108], [126], arguing for a role of age-related increase in ROS leading to decreased proteasomal activity.

Another potential explanation for lower proteasomal activity upon aging is decreased proteasomal content, which was demonstrated in aged human primary epidermal cells at protein level [108]. However, several studies argue for unchanged expression of proteasomes in aged human lymphocytes [118], including T cells [119].

The proteasome is critical for the activation and survival of T cells, and thus impaired proteasomal activity during aging might contribute to age-related T cell defects. Inhibition of the proteasome by bortezomib upon T cell activation leads to accumulation of ubiquitinated proteins, reduced proliferation and induction of apoptosis in human CD4^+^ T cells [127], and total human T cells [128]. This is accompanied by the induction of cell cycle arrest, related to the accumulation of cell cycle inhibitors p21^WAF1/CIP1^ and p27^KIP1^ [127]. Similarly, treatment of T cells with the proteasome inhibitor lactacystin inhibits proliferation, and this cell cycle arrest is accompanied by accumulation of cyclin E and p27^KIP1^, and induces apoptosis in activated and to a lesser extent in resting T cells [129]. Finally, cyclosporin A-mediated proteasome inhibition also leads to accumulation of p27^KIP1^ but induces apoptosis preferentially in resting human CD4^+^ T cells [130]. An age-related decline in proteasome activity of human T cells was also associated with lower expression of the IL-2 receptor following TNF-α stimulation [119], which further impairs T cell activation and survival.

Defects in the induction of the proteasome in CD4^+^ T cells upon TCR stimulation have been associated with a senescent T cell phenotype, characterized by defective proliferation, cytokine production and increased levels of PD-1^+^CD44^hi^ unresponsive CD4^+^ T cells [131]. Confirmatively, expression of the proteasome component Rpn11 upon TCR stimulation was lower in aged CD4^+^ T cells, and most of the Rpn11-low expressing T cells belonged to the senescent PD-1^+^CD44^hi^ CD4^+^ T cell subset [131].

Besides the importance of the proteasome for degradation of proteins, thus maintaining proteostasis, the proteasome is also involved in the generation of antigenic peptides for presentation on MHC class I molecules, critical for activation of CD8^+^ T cells and induction of an immune response [132]. This critically shapes the immune system and dysfunctions in this process also may contribute to pathologic conditions observed upon aging. The exposure of lymphocytes to IFN-γ induces the expression of three facultative catalytic proteasome subunits, β1i/LMP2, β2i/MECL-1, and β5i/LMP7, which are preferentially incorporated into newly assembled proteasome complexes and replace their constitutive homologues, thus forming the so-called immunoproteasomes [133], [134]. A study published by Zanker *et al.* particularly demonstrated that the proteasome composition not only determines the pool of epitopes generated and presented by APCs, but also actively shapes the T cell repertoire contributing to the diversity of CD8^+^ T cell responses [135]. Combining these findings and the observation that proteasomal activity decreases with aging, the narrowing of TCR repertoires observed upon aging [136], [137] may depend in part on defects in proteasomal antigen processing. However, human TCR diversity in naïve CD4^+^ and CD8^+^ T cells declines only moderately during aging, which guarantees an appropriate response to new infections and formation of new memory T cells [136], [137]. Nonetheless, there is evidence for uneven homeostatic proliferation of naïve T cells in older individuals based on large quantities of singular naïve T cell clones, that could lead to perturbations of the T cell repertoire and influence the immune response in aged individuals [136].

Several studies in lymphocytes showed that the proteasome can influence the differentiation and function of T cells. The pathology of the Sézary syndrome is linked to T_H_2 cytokine skewing and an overexpression of GATA3 and the T cell inhibitory molecule CTLA-4, and there is evidence that a defect in proteasomal degradation is the underlying factor triggering this T cell phenotype [138]. Another study by Widjaja *et al*. highlighting the importance of the proteasome for T cell fate decision . demonstrated an influence of the proteasome on differentiation of CD8^+^ T cells into effector or memory T cells during *Listeria monocytogenes* infection [139]. After their first division, pre-effector CD8^+^ T cells (IL-2Ra^hi^CD62L^lo^) showed lower proteasome activity than pre-memory T cells (CD8^+^IL-2Ra^lo^CD62L^hi^) [139]. Consequently, inhibition or activation of the proteasome resulted in increased differentiation into effector and memory-like subsets *in vitro* and *in vivo*, respectively [139]. An age-related decline in proteasome activity could thus contribute to reduced formation of memory T cells, and increased differentiation into effector T cells.

The proteasome is critical for activation of canonical NF-κB signaling, as degradation of the inhibitor IκBα, which sequesters NF-κB in the cytoplasm, is mediated by the proteasome [140]. This enables nuclear translocation of NF-κB und mediates transcription of several target genes associated with activation and cytokine production [140]. Therefore, age-related decline or inhibition of the proteasome is associated with decreased NF-κB activity due to impaired degradation of IκBα and reduced expression of pro-inflammatory cytokines as has been reported in TNF-α stimulated T cells from older individuals [119], PHA-stimulated human T cells treated with proteasome inhibitor bortezomib [128], and in a mouse model of contact hypersensitivity [141]. However, hyperactive NF-κB responses are observed in physiological conditions marked by proteasomal dysfunction, including advanced age [142]. Interestingly, a study from Cullen *et al.* demonstrated increased production of the pro-inflammatory cytokine IL-6, using a modified murine stromal cell line, upon inhibition of the proteasome [142]. This effect was attributed to atypical activation of the NF-κB pathway [142], leading to dissociation of IκBα from NF-κB due to tyrosine phosphorylation without involvement of the proteasome [143], and this process is promoted by oxidative stress [144]. Given the fact, that an increase in oxidative stress, as well as decreased proteasomal activity is observed upon aging, this mechanism could contribute to the development of chronic inflammation with age.

Indeed, oxidative stress leads to the disassembly of proteasomes in yeast and mammalian cells and attenuates ubiquitin-dependent proteolysis [145]. Treatment of human T lymphocytes with the prooxidant buthionine sulfoximine (BSO) resulted in decreased proteasome activity, reduced TNF-α-induced IκBα degradation and NF-κB activity, as well as lower activation-induced proliferation in T cells from young donors, mimicking the results obtained for untreated T cells from old donors [146]. Interestingly, the phenotype in T cells from young donors was reversible by treatment with the antioxidant NAC, while treatment of T cells from old donors with NAC did not increase the proteasomal activity, or activation-induced proliferation [146].

### 4.2 Cytoplasmic enzymes – tripeptidyl peptidases and the calpain-calpastatin system

In addition to the proteasome, other cytosolic degradation systems such as the tripeptidyl peptidases (TPP) and the calpain-calpastatin system (CCS) are implicated in T cell function and in processes of T cell aging.

Tripeptidyl peptidase II is an aminopeptidase with both, exo- and endopeptidase activity, and is implicated in processing peptides for presentation on MHC I molecules, which functions in tandem with the proteasome-ubiquitin protein degradation pathway [147], [148]. Mice with TPP II deficiency show premature cellular senescence in CD8^+^ T cells, associated with an impaired survival upon proliferation and increased basal NF-κB activity, as well as a decreased lifespan and signs for immune cell deficits such as lymphopenia, and early thymic involution, linking TPP II to the maintenance of immune cell function and dysregulation of the immune system to accelarated aging [149]. However, normal development and function of CD8^+^ T cells after immunization with recombinant lentiviral or vaccinia virus vectors was found in another study using TPP II-deficient mice, although expression of MHC I on splenocytes was increased and processing of N-terminal extended model epitopes from LCMV by embryonic fibroblasts was reduced in these mice, arguing for a role of TPP II in both, degradation of MHC I molecules and processing of MHC I epitopes [150].

Autosomal recessive *TPP2* mutations in humans were linked to recurring respiratory or systemic infections, autoimmunity, and neurodevelopmental delay [148]. This was attributed to critical involvement of TPP II in maintaining cellular amino acid levels as demonstrated in murine embryonic fibroblasts and a human neuroblastoma cell line [148]. In human TPP II-deficient T cells, lysosomal biogenesis was upregulated, which was associated with degradation of hexokinase-2 and thus reduced glycolysis, resulting in decreased frequency of IFN-γ^+^ CD4^+^ and CD8^+^ T cells [148]. TPP II deficiency in humans is also associated with the development of early-onset Evans syndrome and, similarly to mice, the T cells of patients with TPP II deficiency show a senescent phenotype, accompanied by a proliferation defect, but, in contrast to the aforementioned study, enhanced perforin and IFN-γ expression [151].

The calpain-calpastatin system (CCS) consists of cytosolic, calcium-dependent cysteine proteases, of which µ- and m-calpain are characterized best, and the constitutively expressed cytosolic inhibitor calpastatin [152]. Calpains have been reported or predicted to cleave and thus regulate a variety of protein functions, including proteins involved in proliferation, cell differentiation, activation, and cell death [153]^, reviewed in^ [154], [155]. The actions of calpains are described as “proteolytic processing”, as they act by cleaving rather than degrading their target proteins [154]. Expression of both, µ- and m-calpain was lower in CD4^+^ and CD8^+^ T cells from old individuals, while calpastatin expression was only lower in CD8^+^ T cells derived from aged human donors compared to T cells from young donors [156]. Calpains show constant activation in resting human T cells and inhibition prevents proliferation after T cell activation without inducing apoptosis [157]. Activation of PBMCs in the presence of calpain inhibitors also resulted in reduced production of T cell effector cytokines, and reduced phosphorylation of phospholipase C γ, Lck and NF-κB in CD4^+^ and CD8^+^ T cells [157], [158]. Reduced expression of calpain as found in T cells from older adults could contribute to the reduced potential of T cell activation observed upon aging, providing yet another example how components of the protein degradation system influence T cell function upon aging.

### 4.3 Autophagy

Upon autophagic degradation, cytoplasmic proteins or organelles are engulfed by autophagosomes, characterized by double membranes, and delivered to lysosomes for degradation [16], [159]. Three different types of autophagy are discriminated, namely macroautophagy, microautophagy and chaperone-mediated autophagy (CMA), distinguished by different cargo size and cargo delivery mechanisms [160]. Autophagy is critical for maintaining cellular homeostasis as it is capable of degrading large protein aggregates and whole damaged organelles [160], [161]. Dysfunctional autophagy is associated with decreased lifespan, reduced healthspan of eukaryotic organisms, and impaired functionality of different cell types [100]. Formation of autophagosomes is mediated by many proteins, most importantly the ULK1 complex, involved in initiation of autophagosomal formation, and autophagy-related proteins Atg3, Atg5, Atg7, Atg10, and Atg16L1 participating in elongation of the emerging membrane [16]. The transcription factor EB (TFEB) acts as a master activator of lysosomal biogenesis, but also autophagy, and is sequestered at the lysosomal membrane by mTORC1 under nutrient-rich conditions [162]. However, upon starvation AMPK activates autophagy through inhibition of mTORC1 and by activating phosphorylation of ULK1, which leads to formation of autophagosomes [163].

Autophagic flux and the expression of autophagy-related proteins gradually decline with age in various organisms [99], [100]. Expression of core autophagy genes and proteins is reduced upon aging in *Drosophila melanogaster* [164], brains of mice [165], rats [166], and humans [167]. Defects in autophagy based on mutations or artificial disturbance of Atg genes lead to decreased lifespan in *C. elegans* [168], *Drosophila melanogaster* [169], and is lethal or impairs health and lifespan in mice ^reviewed in^ [170]. Additionally, tissue-specific knockout of Atg proteins in mice leads to age-associated defects such as accumulation of dysfunctional organelles and proteins, disorganized mitochondria [171], [172] and endoplasmic stress ^reviewed in^ [173], [174], while increasing autophagy by rapamycin treatment [175], [176], or overexpression of autophagy genes [177], [178] promotes clearance of damaged and aggregated proteins. In T cells, autophagy activity has been demonstrated to decline with aging in murine CD8^+^ T cells [179] and human CD4^+^ and CD8^+^ T cells [180], [181], [182], [183]. This affects differentiation, maintenance and function of naïve, effector and memory T cells.

Reduced frequencies of circulating naïve CD8^+^ T cells are considered a hallmark of aging in humans [6], [7], [8], [10], while the population of naïve CD4^+^ T cells is reported to be more stable upon aging [7], although several studies also demonstrated reduced frequencies of naïve CD4^+^ T cells in PBMCs from older adults [6], [10], [11]. One important step in the development of naïve T cells is the degradation of mitochondria and a switch from oxidative phosphorylation to glycolysis, which is critical for evolving from single positive thymocytes into mature peripheral naïve T cells [160], [184]. Naïve Atg7-deficient T cells showed increased mitochondrial content, which results in increased ROS accumulation and higher rates of cell death [185]. Autophagy is critically involved in the maintenance of T cell populations in general, as evidenced by reduced numbers of both, naïve and total peripheral T cells in mice with autophagy-deficient T cells [185], [186], [187], [188], [189], [190]. Autophagy regulates the proliferation and survival of T lymphocytes through selective degradation of the cell cycle inhibitor CDKN1B [191] and pro-apoptotic proteins [188], although inhibition of the proteasome also delayed the degradation of CDKN1B [191]. Additionally, CD8^+^ and CD4^+^ autophagy-deficient peripheral T cells show increased apoptosis upon activation [186], [188], [189].

Additionally to reduced frequencies of naïve T cells, impaired formation of memory T cells in response to primary infection or vaccination is considered a hallmark of aging [192], resulting in decreased capacity of the immune system to fight pathogens and higher death rates in older adults [1]. Impaired generation of memory T cells, which is observed in aged mice after primary challenge of the organism through infection or immunization, points towards defects in primary T cell responses [193], [194]. The first step to memory formation is activation through TCR engagement. This leads to upregulation of macroautophagy in murine T cells [186], murine and human CD4^+^ T cells [195], [196], [197], [198], [199] and human CD8^+^CD28^+^ lymphocytes [200]. Supportively, defects in macroautophagy inhibit T cell activation [196] and reduce peripheral T cell survival [186], [187]. A recent study by Zhou *et al.* uncovered a mechanism underlying defective CD4^+^ T cell activation and proliferation in absence of autophagy [201]. Atg7-deficient T cells showed excessive surface expression of IL-7Rα, which is normally degraded by autophagy upon T cell activation [201]. This results in sequestration of the common gamma chain, thus impairing IL‐2R assembly and downstream signaling, which is critical for activation and proliferation of T cells [201]. Autophagy induction via JAK signaling and common gamma chain receptors has also been demonstrated in TCR-stimulated murine CD4^+^ T cells by Botbol *et al.* [197]. Interestingly, in this study mTOR inhibition by rapamycin did not influence autophagy induction in response to TCR stimulation, arguing for autophagy induction in an mTOR-independent manner [197]. The signaling mechanisms leading to activation of autophagy upon TCR stimulation have not been completely elucidated but it has been proposed that in addition to JAK signaling [197], [201] activation of MAPK (JNK1/JNK2) leads to activation of FOXO1 or release of Beclin-1 thus inducing autophagy, although this has not been demonstrated in T cells so far ^reviewed in^ [160], [202]. The importance for autophagy in activation of T cells is further highlighted by a study from Mocholi *et al.*, demonstrating the accumulation of TCR inhibitory phosphatase PTPN1 in Atg7-deficient CD4^+^ T cells, and decreased activation of human CD4^+^ T cells upon TCR stimulation if autophagy is dampened by siRNA knockdown targeting Atg5 [203]. Interestingly, a study by Xu *et al.* challenges previous studies which demonstrated autophagy activation in response to TCR stimulation: In transgenic P14 mice infected with LCMV, levels of LC3B and p62 protein were increased in CD8^+^ T cells five days after infection, while autophagic flux was highest eight days after infection, arguing for inhibition of autophagy in earlier stages of antigen-induced T cell activation [204]. Further studies will be necessary to evaluate how and when autophagy is activated in response to TCR stimulation or antigen-induced activation.

However, decreased memory responses upon aging could also result from impaired maintenance of long-lived T memory cells. Human memory T cells have an extremely long half-life [205], and a very low division rate [206]. Upon aging, the majority of studies report increased frequencies of memory and effector T cells ^reviewed in^ [207], albeit with decreased functional responsiveness to new antigens [194], and the severely reduced response toward vaccination detected in older adults could be influenced by age-related defects in macroautophagy [179]. Kang *et al.* reported impaired maintenance of memory T cells in older individuals after initial normal formation of memory responses following influenza vaccination [208]. Confirmatively, naïve CD4^+^ T cells from aged mice generated fewer central memory T cells in response to OVA immunization compared to naïve CD4^+^ T cells from younger mice, which was linked to the presence of defective mitochondria and reduced autophagy [209]. Mice bearing Atg7-deficient T cells showed impaired memory T cell formation following viral infection, which was accompanied by increased apoptosis and mitochondrial damage [179]. Deletion of Atg5 in T cells at the mature state decreased the survival of CD8^+^, but not CD4^+^ T cells, and hindered the sustained production of anti-OVA IgG antibodies following immunization, also arguing for a defect in immunological memory formation [190]. Subsequent analysis postulated decreased survival of memory T cells differentiated *in vitro* from splenocytes of Atg5^f/f^ dLck-cre mice compared to memory T cells from WT splenocytes, due to increased presence of damaged mitochondria and lipid content [190], which implicates a pivotal role of autophagy in cellular maintenance and regulation of metabolism. Consistently, knockout of Atg5 in CD8^+^ T cells during the effector phase of acute influenza infection impaired the initial effector response, caused by higher production of ROS leading to apoptosis, but also prevented effective formation of memory T cells [210]. Furthermore, Atg7-deficient T cells were unable to differentiate into long-lived memory T cells following LCMV infection and mice with Atg7-deficient CD8^+^ T cells were unable to control LCMV viral burden [204], which mimics the impaired capacity of elderly people to effectively clear microbial infections. Similarly, the immune response against *Listeria monocytogenes* infection was impaired in mice with Atg3-deficient T cells [191].

One factor contributing to susceptibility of naïve and memory T cells to dysfunction upon autophagy inhibition is their metabolic demand. Longer-lived quiescent T cells rely on fatty acid oxidation and oxidative phosphorylation (OXPHOS), which is supported by autophagy [211], [212]. Interestingly, T_regs_ were also shown to preferentially use fatty acid oxidation and OXPHOS, while effector T cells depend on glycolysis [211], [213].

Thus, it is not surprising that autophagy is critical for the maintenance of T_regs_. Frequencies of CD4^+^FoxP3^+^ T cells are lower in mice with Vps34-deficient T cells [214] and deletion of Atg7 in murine T_regs_ leads to decreased lineage stability and reduced survival of those cells [215]. Basal autophagy levels are also higher in T_regs_ compared to naive CD4^+^ T cells and effector T cells, and active autophagy limits mTORC1-dependent c-Myc expression and function, which would induce glycolysis rather than oxidative phosphorylation, and thus stabilizes the T_reg_ lineage [215], [216]. T_reg_-specific deletion of Atg7 favors the activation and expansion of effector T cells, and leads to the development of multi-organ inflammation, especially in the skin and gastrointestinal tract of aged mice [216]. A similar phenotype was observed upon deletion of Atg16L1 in T_regs_, resulting in loss of regulatory T cells in the mouse intestine, increased frequencies of T_H_2 cells, aberrant expression of IFN-γ and IL-17 by T_regs_, and intestinal inflammatory responses [217]. This phenotype was also attributed to higher expression of c-Myc, and a metabolic shift towards increased glycolysis and oxidative phosphorylation but decreased fatty acid oxidation [217]. Supportively, mutations in the gene encoding Atg16L1 were identified as a risk factor in humans for developing Crohn’s disease [218]. Similarly, deletion of Vps34 in T cells, also resulted in gastrointestinal inflammation in mice [214]. In total, age-dependent decrease in autophagic activity could contribute to increased chronic inflammation observed upon aging, called inflamm-aging [23].

Counterintuitively, in humans, the number of CD4^+^ regulatory T cells does not decline, but increases with age in PBMCs of humans [219], [220], [221], and in spleens and lymph nodes, but not PBMCs of mice [219], [222]^, reviewed in^ [223]. There is no evidence for diminished function of these cells upon aging, as human T_regs_ from old and young donors/mice showed similar or higher suppressive function [219], [220]. Therefore, increased frequency of functional T_regs_ potentially contributes to age-related increased cancer rate [222] and increased susceptibility to infection [224]. However, this contrasts with the finding of age-associated inflamm-aging [23]. A study from Elyahu *et al.* resolved this seeming contradiction. In depth characterization of CD4^+^ T cells from young and old mice revealed the accumulation of both, activated T_regs_ with strong immunosuppressive potential, and cytotoxic pro-inflammatory CD4^+^ T cells, potentially contributing to inflammation, with age [55].

Recently, an influence of autophagy on the IL-9-producing T_H_9 subset, which mediates effective anti-tumor activity [225], [226], but is also implicated in the pathology of chronic inflammatory diseases, including ulcerative colitis [227], [228], has been suggested. The differentiation into T_H_9 cells is inhibited by autophagy through selective degradation of the master transcription factor PU.1 [229]. Activated Atg3- and Atg5-deficient T cells differentiated *in vitro* into T_H_9 cells show increased production of IL-9 compared to their WT counterparts [229], and mutations in core autophagy genes increase the risk for development of inflammatory bowel disease (IBD) in humans [218]. Interestingly, naïve CD4^+^ T cells from aged individuals were biased towards T_H_9 differentiation when cultured under polarizing *in vitro* conditions, but also after TCR activation under nonpolarizing conditions [230]. Thus, decreased autophagic flux in aged individuals could contribute to the bias towards T_H_9 polarization of T cells, favoring a pro-inflammatory environment and inflamm-aging, although this has to be confirmed in upcoming studies.

Recent findings further linked defective autophagy in CD4^+^ T cells directly to inflamm-aging. Bharath *et al.* demonstrated that activated human CD4^+^ T cells from older individuals showed higher production of proinflammatory T_H_17-associated cytokines compared to young donors’ CD4^+^ T cells [183]. This was due to a defect in autophagy that resulted in increased mitochondrial mass, proton leak, and ROS production [183]. The phenotype could be mimicked by knockdown of Atg3 in CD4^+^ T cells of young donors and was reversed by treatment with autophagy-inducing agent metformin [183], [231]. These findings are supported by a murine study showing increased production of IL-17 and IL-6, and a bias towards induction of T_H_17 cells in activated CD4^+^ T cells from old mice [232], although the contribution of autophagy was not investigated. However, all CD4^+^ peripheral T cell subsets, including T_H_17 cells, exhibit significantly increased susceptibility to cell death after deletion of autophagy-related genes Beclin-1 [188], or Vps34 [233]. Human CD4^+^ T cells from older individuals showed enhanced basal activity of NF-κB and higher transcription of genes coding for pro-inflammatory cytokines, which was ameliorated by both, inhibition of PI3K and induction of autophagy through rapamycin treatment [234]. Interestingly, autophagic flux and IFN-γ production was higher in activated CD4^+^ T cells from the offspring of families with exceptional longevity compared to age-matched controls, linking increased autophagic activity in those cells to increased life- and healthspan [198]. Supporting the beneficial effect of autophagy, constitutive activation of autophagy achieved by expression of a mutant Beclin-1 variant in mice results in increased life- and healthspan [235].

Degradation of proteins by CMA is mediated by recognition of a signal peptide (KFERQ) by Hsc70, which initiates targeting to lysosomes and translocation into lysosomes through interaction with CMA receptor LAMP-2A [236], [237]. CMA activity was demonstrated to decrease with aging in rat liver [238], [239]. Potential contributing factors to this decrease are altered lipid composition of the lysosomal membrane [239], [240], and the reduced abundance and stability of LAMP-2 at the lysosomal membrane [241], [242]. Knockout of LAMP-2 in mouse liver also resulted in altered proteostasis and hepatic dysfunction in mice with age [243]. Conversely, activity of CMA is maintained in aged animals at comparable levels as in young mice if abundance of LAMP-2 is kept at a constant level through genetic modification [241]. CMA activation upon TCR engagement has been demonstrated in murine CD4^+^ T cells through upregulation of LAMP-2A and targeted degradation of inhibitory TCR regulators Itch and RCAN1 [244]. Knockdown of LAMP-2A expression using shRNA or knockout of LAMP-2A in mice led to impaired T cell activation, reduced proliferation and cytokine production upon stimulation or *Listeria monocytogenes* infection [244]. Interestingly, LAMP-2A expression was lower in CD4^+^ naïve T cells from aged mice (protein) and older humans (mRNA) [244], which could lead to impaired T cell proteostasis and function upon aging.

## 4.4 Lysosome

Lysosomes are dynamic central degradation organelles characterized by acidic intraluminal pH, which is maintained by the vacuolar ATPase (V-ATPase) [245]. They contain a set of various hydrolases for the degradation of proteins, lipids, and nucleic acids which exhibit the highest activity at low pH values, also to prevent damage to intracellular components upon lysosomal leakage [104], [236]. The lysosomal cargos comprise extracellular and surface materials incorporated after endocytosis as well as intracellular materials delivered via autophagy [246]. However, lysosomes are also involved in processes other than waste disposal, such as stress resistance, programmed cell death as well as cell development and differentiation [236]. They store nutrients, metabolites and ions such as calcium and iron [236]. Furthermore, several studies point towards a critical role of lysosomes in the regulation of cellular responses to nutrient availability by functioning as a signaling hub for various molecules including the mTORC1 complex [247].

There is accumulating evidence supporting a general decline in lysosome function with age ^reviewed in^ [248], as well as age-dependent alterations in size, number and lysosomal content [249]. Furthermore, disturbances of the lysosomal membrane and lysosomal proteins are linked to leakage of proteolytic enzymes upon aging, which triggers damage responses and cell death ^reviewed in^ [250], [251]. Lysosomal defects are associated with the formation of lipofuscin, an insoluble intralysosomal polymer, which consists of aggregated oxidized proteins further reacting with cellular components [252], [253], [254]. The effects of lipofuscin on the cell are unknown, but lipofuscin may be involved in the production of oxidants leading to post-translational modifications of cellular components [255] or inhibition of the proteasome [256]. Lipofuscin accumulation is linked to caspase-3 activation in senescent human dermal fibroblasts [255], and promotes lysosomal membrane disruption leading to activation of the NLRP3 inflammasome and induction of necroptosis in retinal pigment epithelial cells in a mouse model of Stargardt and dry age-related macular degeneration [257]. Protein aging itself is also implicated in incapacitating the degradative capacity of lysosomes ^reviewed in^ [248].

Aging is generally associated with an increase in cellular ROS leading to conformational changes in TCR components, decreased DNA replication, and lower rates of T cell proliferation, thus affecting T cell responses [97]. One major factor for increased concentration of ROS upon aging is reduced mitophagy, a selective form of autophagy [258], [259], leading to accumulation of damaged mitochondria which accelerates aging [260], [261]. The lysosome is critically involved in degradation of dysfunctional mitochondria via mitophagy [259]. Lysosomal dysfunction, for example due to impaired lysosomal acidification [262], is linked to an increase in damaged mitochondria. Accumulation of lipofuscin due to disturbed lysosomal function further increases ROS production, which results in a feedback loop of dysfunctional mitochondria and lysosomes [263]. Mitochondrial turnover is also impaired in autophagy-deficient T cells [185], [264], leading to functionally altered mitochondria, increased ROS production and higher rates of cell death [185], [264]. Bektas *et al.* detected accumulation of immature autophagosomes containing mitochondria in CD4^+^ T cells from aged healthy individuals [181], arguing for a defect in mitophagy in T cells from older persons. Mitochondrial stress leads to the release of danger associated molecular patterns (DAMPs) resulting in activation of innate immune cells and production of proinflammatory cytokines [100], which potentially contribute to inflamm-aging.

Malfunctions and reduced subunit expression of the V-ATPase are reported during cellular aging, leading to de-acidification, as well as reduced protein degradation, and defects in V-ATPase function are implicated in the pathogenesis of age-related neurodegenerative diseases [249], [265]. As mitochondria and lysosomes are connected, cells lacking V-ATPase subunits also display mitochondrial dysfunctions attributed to cellular iron deficiency [266]. Gradual decline of the vacuolar acidity leading to mitochondrial dysfunction has also been reported during replicative aging of *S. cerevisiae* [262], and an increase in lysosomal pH has been defined as a hallmark of aging in *C. elegans* leading to disturbed proteostasis and the end of reproduction [267]. However, so far there are no studies on the role of V-ATPase or iron levels in aged lymphoid immune cells.

Impaired lysosomal activity observed in naïve CD4^+^ T cells from older adults was described to limit proliferation in response to T cell activation, due to inefficient lysosomal degradation of the inhibitory receptor PD-1 [15]. The receptor PD-1 is expressed upon activation of T cells, and binding of the PD-1 ligand (PD-L1) expressed on APCs triggers inhibitory signaling, thus attenuating T cell responses [268]. Upon ligation, PD-1 is internalized, sorted into endosomes and directed for lysosomal degradation, leading to reduction of the inhibitory signal after initial upregulation upon activation [15]. Jin *et al.* found sustained activation of mTORC1 on late endosomes in T cells from old human donors, which prevented PD-1 from lysosomal degradation, thus hindering T cell activation and expansion [15]. PD-1 was also found to be highly expressed on exhausted murine CD8^+^ T cells upon chronic LCMV infection, which correlated with reduced glucose uptake and metabolic use and impaired formation of antigen-specific effector cells [269]. Therefore, reduced degradation of PD-1 due to lysosomal dysfunction could contribute to impaired T cell activation and inefficient response to infections upon aging.

The lysosomal surface provides the platform for mTORC1 as a critical signaling hub in nutrient sensing and regulation of cellular metabolism and growth [247], [270]. Under nutrient rich conditions, high concentrations of amino acids present in the lysosomal lumen lead to recruitment of mTORC1 to the lysosomal surface [271], [272]. This leads to sequestration of the transcription factor TFEB, a master activator of lysosomal biogenesis and autophagy [162], at the lysosomal surface through inhibitory phosphorylation by mTORC1 [273]. Upon starvation, mTORC1 is inactivated, which abrogates the inhibitory phosphorylation of TFEB and thus initiates nuclear translocation of TFEB and transcription of genes involved in lysosomal biogenesis and autophagy [247], [274]. The activation of mTORC1 favors differentiation of effector T cells over the formation of memory or follicular T helper cells [48], [275].

A recent study revealed late endosomes, also called multivesicular bodies (MVBs), as an alternative platform for mTORC1 signaling in T cells [15]. MVBs arise from endosomes, which incorporate proteins destined for lysosomal degradation in intraluminal vesicles (ILVs) through invagination of the limiting membrane [276], [277]. MVBs fuse with lysosomes, which leads to degradation of ILVs and their respective cargo [276], but also provides molecules for maintenance and generation of lysosomes, such as lysosomal membrane proteins or hydrolases [277]. Thus, lysosomal activity is a major determinant of MVB turnover, and, consequently, knockdown of TFEB in murine CD4^+^ T cells leads to accumulation of MVBs [278]. Recently, reduced expression of the transcription factor FOXO1, which promotes lysosome and autophagy function through induction of TFEB, was measured in stimulated naïve CD4^+^ T cells from aged human donors, which consequently led to increased accumulation of MVBs accompanied by sequestration of glycogen synthase kinase 3β (GSK3β) [278]. Lysosome-inhibited CD4^+^ T cells also displayed decreased intracellular protein turnover, increased cell size and enhanced glycolytic activity, which promoted effector T cell differentiation [278]. Consequentely, impaired lysosomal function, observed in mice with CD4^+^ T cells deficient for the mitochondrial transcription factor Tfam, was connected to exacerbated inflammatory responses in a DSS colitis model and promoted the *in vitro* differentiation of IFN-γ-producing CD4^+^ T cells from naïve T cells [279]. In healthy cells, mTORC1 signaling is regulated in a negative feedback loop, as mTORC1 activity leads to downregulation of lysosomal biogenesis, thus abolishing the platform for mTORC1 [280]. However, the study by Jin *et al.* points towards increased usage of late MVBs as a platform for mTORC1 signaling in activated T cells from older adults, which results in a feedforward loop [15], as sustained strong mTORC1 signaling leads to inhibition of lysosomal biogenesis, followed by accumulation of MVBs, which themselves serve as platform for mTORC1 activity [15]. Therefore, lysosomal dysfunction upon aging promotes accumulation of MVBs and constitutive mTORC1 expression, which favors development of proinflammatory CD4^+^ effector T cells.

Accumulation of MVBs upon lysosomal inhibition also leads to a shift in the trafficking route of MVBs towards the plasma membrane in different cell types, including T cells, cumulating in increased secretion of exosomes [278], [281]. TFEB enhances lysosomal exocytosis by increasing the amount of lysosomes near the plasma membrane and increasing intracellular calcium concentrations through activation of the lysosomal calcium channel MCOLN1 [282]. In CD8^+^ T cells, the process of lysosomal exocytosis is critical for efficient cytotoxic function as those compartments contain effector molecules such as perforin, granzymes, and inhibiting or apoptosis-inducing surface molecules [283], which are delivered to the plasma membrane. In aging CD4^+^ T cells, reduced lysosomal activity due to decreased levels of FOXO1 was connected to increased exocytosis of secretory lysosomes containing granzyme B [278]. Incubation of B cells with exosomes collected from activated CD4^+^ T cells from old donors induced caspase-3 activity and apoptosis, which directly links lysosomal dysfunction upon aging to increased cytotoxic effector functions [278]. In contrast, knockout of TFEB in mice resulted in decreased production of intracellular IFN-γ and perforin by activated CD8^+^ T cells, while intracellular levels granzyme B remained unchanged [182]. This argues for a critical role of lysosomes for CD8^+^ T cell effector function, but its role in driving inflamm-aging needs to be further explored.

## 5 Enhancing proteostasis in aging T cells – an anti-aging approach?

**Fig. 3:**
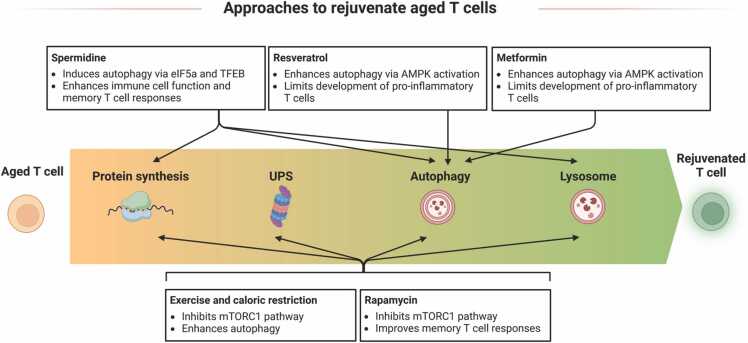
**Different approaches to rejuvenate aged T cells.** Several interventions have been proposed to either prevent T cell aging or rejuvenate aged T cells. Those include administration of pharmacological compounds like spermidine, resveratrol, metformin, and rapamycin, but also physiological interventions like exercise and caloric restriction. Those act on different factors of the proteostasis network, leading to improved or altered T cell function and rejuvenation of T cells.

There is no doubt that filling the gaps in our knowledge of how to maintain a healthy proteome with age will lead to new therapeutic approaches. Importantly, if rejuvenation via these pathways is successful, this strongly suggests that they are worth studying, and not just epiphenomena of aging. Fig. 3 contains a summary of agents and approaches which are currently discussed for rejuvenation of T cells.

Briefly and as reviewed elsewhere in detail [284], autophagy-inducing pharmacological agents, as well as exercise and caloric restriction are linked to extended life- and healthspan in various organisms [95] and are therefore promising candidates for future therapeutic approaches [285], also for rejuventation of T cells. Several of those interventions act through inhibition of the mTORC1 pathway, which negatively regulates autophagy [273], [286], [287]. mTORC1 also prevents nuclear translocation of the transcription factor EB (TFEB), which acts as a master transcription factor promoting expression of lysosomal and autophagic genes and therefore catabolic processes [162], [273], [288]. Inhibition or downregulation of mTORC1 results in extended lifespan and healthspan in various organisms ^rewieved in^ [248]. Caloric restriction and exercise have been demonstrated to extend lifespan or healthspan through induction of autophagic flux or autophagy genes in several organisms, including rodents [289], primates [290] and humans [291], [292].

Over the last years, the autophagy inducing metabolite spermidine has gained increased attention due to its cardioprotective, neuroprotective and immune resilience promoting effects and lifespan-extending properties [174], [293], [294]. However, spermidine levels drop with age in various cells, including T cells [182], and thus, supplementation of spermidine is being discussed as a therapeutic intervention to support healthy aging [294]. Zhang *et al.* demonstrated, that spermidine post-translationally modifies the translation factor eIF5A, which subsequently controls autophagy and transcription factor TFEB expression in primary B cells [295]. Similarly, spermidine was shown to rejuvenate human T cells, as treatment of CD8^+^ T cells from old donors restored autophagy via the same pathway as in B cells, resulting in increased functionality of those cells [182]. Puleston *et al.* illustrated that the autophagy-inducer spermidine can restore CD8^+^ T cell memory response function in aged mice [179]. Spermidine also contributes to the maintenance of proteostasis as evidenced by increased degradation of misfolded proteins upon spermidine supplementation in a mouse model of mild cognitive impairment [296]. Resveratrol, another autophagy-inducing agent, reduces the pro-inflammatory cytokine profile observed in aged mice [297]. Similarly, treatment of mice with rapamycin, inhibitor of mTOR, extends their lifespan [298], [299], and rapamycin was also demonstrated to increase the generation of CD8^+^ memory T cells in a murine LCMV infection model [48].

Further research and rescue experiments or rejuvenation of aged T cells are needed to elucidate which of these pathways are important in aging, and can be targeted by drugs. Importantly, as any pathway identified that affects the healthy proteome in aging will likely have an effect globally, human T cells are actually a great cell type to test these, as they are easily accessible in blood, available in high numbers and genes of interest can now be specifically targeted with CRISPR-Cas systems. Lastly therapies can be tested by interrogating the improvement of T cell function to a vaccine or another safe immune challenge.

## 6 Conclusions and perspectives

Overall, the studies presented in this review indicate that impaired proteostasis contributes to the aging phenotype of T cells similar as seen in cells of other tissues but many areas still need a more detailed analysis in T cells. Additionally, some of the changes observed in aging cells, and aging T cells in particular, could rather be protective than maladaptive, which has to be further elucidated. Most of the studies also did not address the potential of different degradation pathways to compensate for each other, which was demonstrated in various cell types ^reviewed in^ [300], [301]. In many studies only snap shot measurements of phenotypes comparing T cells from old and young were carried out. This might not be the best way to understand what is cause and what is consequence, as cells have the time and ability to adapt to an age-related decrease, or an artificial knock-out in experimental models, of a certain pathway. So superior to snap shots, a kinetic analysis over the life time of the organisms, and even better including centenarians, would be beneficial in this respect. Also, in T cells little attention has been given to the accumulation of protein aggregates, which are a major consequence of impaired proteostasis in other cell types such as neurons. With new technologies of detecting aggregates such as with flow cytometry [302], this question should be easily addressed. Furthermore, it has not been answered if proteostasis is particularly at peril in long-lived quiescent cells such as naïve T cells or memory T cells, who divide little and cannot pass on or dilute out their debris to their daughter cells. In future studies therefore, particular attention should be given to subpopulations of T cells. In human, studies were often performed in bulk PBMCs rather than in isolated subpopulations losing the granularity that could be achieved in the specific well studied T cells. In addition, it is unresolved whether the asymmetric segregation of unfolded proteins, protein aggregates and other unwanted material to short lived daughter cells is part of the T cells’ arsenal to combat aging.

Taken together, we know a fair amount about the pathways that maintain proteostasis in T cells, but we know too little about whether these matter in aging, to be able to predict if targeting them would be beneficial.

## Declaration of Competing Interest

The authors declare no competing financial interests.
